# A feasibility study of noninvasive prenatal diagnosis in facioscapulohumeral muscular dystrophy type 1 in a Chinese family

**DOI:** 10.3389/fgene.2022.1046096

**Published:** 2022-10-25

**Authors:** Yayun Qin, Hui Xu, Jingmin Yang, Yiming Wu, Hui Li, Bo Wang, Lijun Liu, Ding Ren, Runhong Xu, Manman Li, Chengcheng Zhang, Jieping Song

**Affiliations:** ^1^ Medical Genetics Center, Maternal and Child Health Hospital of Hubei Province, Wuhan, Hubei, China; ^2^ Shanghai We-Health Biomedical Technology Co., Ltd, Shanghai, China; ^3^ Key Laboratory of Birth Defects and Reproductive Health of National Health and Family Planning Commission (Chongqing Key Laboratory of Birth Defects and Reproductive Health, Chongqing Population and Family Planning, Science and Technology Research Institute), Chongqing, China; ^4^ State Key Laboratory of Genetic Engineering, School of Life Sciences, Fudan University, Shanghai, China

**Keywords:** haplotype, cell-free fetal DNA, BioNano optical mapping, noninvasive prenatal diagnosis, facioscapulohumeral muscular dystrophy type 1 (FSHD1)

## Abstract

**Objective:** To demonstrate the feasibility of haplotype-based noninvasive prenatal diagnosis of Facioscapulohumeral Muscular Dystrophy type 1 (FSHD1).

**Methods:** Bionano optical mapping was used to identify the D4Z4 structural variation of the genomic DNA sample from the proband affected with FSHD1. In addition, based on the technique of next generation sequencing, the pathogenic haplotype was determined by using trio strategy through genotyping his parents, and also fetal inheritance of paternal haplotypes was then deduced using the Hidden Markov Model.

**Results:** Bionano optical mapping analysis revealed that the proband has only three D4Z4 repeats left in the 4q35 chromosomal region and a disease-permitting 4qA haplotype. The other normal allele of the proband contains 29 D4Z4 repeats and also a 4qA haplotype. The noninvasive cell-free fetal DNA (cffDNA)-based haplotype analysis suggested that the fetus inherited the pathogenic allele from his father and thus was predicted to be affected by FSHD1. In addition, Bionano optical mapping also demonstrated the presence of the pathogenic allele in the fetus by interrogating the genomic DNA from the amniotic fluid cells.

**Conclusion:** Our study showed the cffDNA-based haplotyping was feasible for the noninvasive prenatal diagnosis of FSHD1, which is able to provide earlier testing results with a lower risk of miscarriage and infection than invasive techniques.

## Introduction

Facioscapulohumeral muscular dystrophy (FSHD) is one of the most common inherited muscular dystrophies with an estimated prevalence of 1/20,000 (He et al., 2018). Progressive decreases in the strength of facial, shoulder girdle, ankle dorsiflexors, and proximal leg muscles are the typical clinical symptoms of FSHD (Mul et al., 2016). Two subtypes of FSHD, FSHD1 and FSHD2, have been distinguished based on their different underlying genetic models. FSHD1 patients carry a heterozygous pathogenic allele consisting of a contracted D4Z4 repeat array and an immediately following 4qA haplotype in the subtelomeric chromosomal region 4q35. The D4Z4 repeated region contains 11 to 100 copies of a 3.3 kb repeated element in normal individuals. However, in FSHD1 patients, the copy numbers are decreased to 1 to 10 due to the large deletions in the D4Z4 repeat array (Lemmers et al., 2010). The less common form of FSHD, FSHD2, is due to the mutations in structural maintenance of chromosomes flexible hinge domain containing 1 (*SMCHD1*) gene that occurs frequently coexisting with a 4qA haplotype. On the premise of 4qA haplotype, either the reduction of D4Z4 repeats or the insufficiency of SMCHD1 protein could lead to an abnormal expression of the Double Homeobox Protein 4 (*DUX4*) gene, which causes a pathological injury of muscle cells (Rickard et al., 2015).

FSHD is characterized by high clinical heterogeneity and progressive muscular dystrophy. Currently, there is few clinical trials of promising therapies and no effective treatment available for FSHD (Oliva et al., 2019). Thus, prenatal diagnosis is really necessary for most FSHD families to greatly reduce the risk of inheriting pathogenic variants. Due to the extreme length and sequence repeatability of the D4Z4 repeats, the genetic diagnosis of FSHD1 is exceptionally challenging. In the traditional invasive prenatal diagnosis of FSHD1, genomic DNA (gDNA) of the fetus is usually obtained from biological samples of chorionic villus or amniotic fluid, then the gDNA is tested using the methods of pulse field gel electrophoresis, Southern blot and molecular combing (Vasale et al., 2015; Nguyen et al., 2017). A method, combination Bionano optical mapping (BOM) and karyomapping, has been proved to greatly improve the efficiency of invasive prenatal testing for FSHD1 (Zheng et al., 2020). However, there is a small risk of miscarriage and infection still cannot be absolutely avoided (Salomon et al., 2019; Vincenten et al., 2022). The application of cell-free fetal (cffDNA) analysis of maternal plasma in noninvasive prenatal diagnosis (NIPD) can avoid the risk of miscarriage and infection caused by chorionic villus biopsy and amniocentesis. Therefore, NIPD is almost no risk for the pregnant women and fetuses, and has been gradually applied in fetal aneuploidy, monogenic diseases, sex-linked genetic diseases, and so on (Xu et al., 2015; Drury et al., 2016; Guseh, 2020). In recent years, haplotype-based NIPD has been reported to be able to deduce the inheritance status of variants in fetuses accurately (Hui et al., 2017; Ma et al., 2017; Chen et al., 2020). However, NIPD of FSHD1 has not yet been reported.

In this study, we reported a case of Chinese family with typical symptoms of FSHD. Firstly, we excluded the *SMCHD1* gene variation by whole exome sequencing, and demonstrated the structural variation of the D4Z4 repeated sequence in the proband (the fetus’ father) through BOM. Then we found the consistent copy number variation of the D4Z4 repeats in cffDNA of the pregnant woman. Finally, we verified the validity of cffDNA results *via* detecting the amniotic fluid DNA sample. All of the results suggested that the fetus inherited the pathogenic allele, an extremely contracted D4Z4 repeat region followed by a disease-permitting 4qA haplotype, from the proband and thus would likely be affected. Our work provides an earlier and safer solution for the prenatal diagnosis of FSHD1 in families with FSHD1 genetic history.

## Methods

### Participants recruitment and clinical examinations

Two individuals (I-2, II-1), who were suspected of having facioscapulohumeral muscular dystrophy, and their family members were recruited from Hubei Material and Child Health Hospital in this study. The participants underwent a series of related musculoskeletal tests including creatine kinase isoenzyme measurement (CK-MB) and electromyography. For genetic diagnosis, 2 ml of peripheral blood were collected from the proband and his parents, and 5 ml of peripheral blood was collected from his wife, as well as 5 ml fetal amniotic fluid at 19 weeks of pregnancy. All participants signed informed consents. This study was approved by the Ethics Committee of the Maternal and Child Health Hospital of Hubei Province.

### Target capturing and sequencing

The target capture panel (Twist bioscience) contains common Single Nucleotide Polymorphisms (SNPs) localized in 5 Mb upstream and downstream flanking regions of D4Z4 repeats. SNPs were screened from the 1000 Genomes Project, with a minor allele frequency threshold of >30% in Beijing Han population, resulting in 567 SNPs loci selected with a median inter-loci distance of 8.4 kb. In addition, 1,262 common SNPs scattered on autosomes were also included in the panel for calculating fetal fraction and sequencing error rate. Targeted enrichment of the gDNA regions was performed according to the custom panel hybridization protocol (Twist Bioscience, United States ).

gDNA was extracted from peripheral blood using a Nucleic acid extraction automatic system (Enlighten Biotech, China), Cell-free DNA (cfDNA) was extracted from maternal plasma using a cfDNA Extraction Kit (DK607, LifeFeng, China). The Hieff NGS^®^ OnePot DNA Library Prep Kit (12203ES96, Yeasen, China) and Hieff NGS^®^ MaxUp II DNA Library Prep Kit (12200ES96, Yeasen, China) were used for library construction for gDNA (300 ng) and cfDNA (20 ng), respectively. The fragment size of the libraries was detected using Qsep100 (BIOptic, China), and the concentration was quantified using Qubit 4.0 (Thermo, United States ). Then, 400 ng of the cfDNA library and 100 ng of the gDNA library were mixed and used for target region capture following the manufacturer. s instructions. The captured library was quantified by Qubit 4.0 (Thermo, United States ) and analyzed by Qsep100 (BIOptic, China). Finally, the postcapture library was sequenced in 100-bp paired-end mode on MGISEQ-T7 sequencing platform (MGI-tech, China). After base calling, depth of mapped reads on target region were >40X and >300X for gDNA and cfDNA samples, respectively.

### Bionano optical mapping

According to the protocol of Gosden (GOSDEN, 1983), we collected the proband’s peripheral blood and the amniotic fluid cells (at 19 weeks of pregnancy) for primary cell culture. About 1.5 × 106 cells were collected and their high molecular weight DNA was extracted for diagnosis and invasive prenatal detection respectively using Bionano Prep SP Blood and Cell Culture DNA Isolation Kit (80,030, Bionano Genomics, United States ). DNA labelling was performed following manufacturer’s guidelines (Bionano Prep Direct Label and Stain (DLS) Protocol, Bionano Genomics, #30206). The labelled DNA was loaded on the Saphyr chip, with each sample generating over 500 Gb data. Data were analyzed and visualized using Bionano Solve (Version 3.7) software.

### Noninvasive prenatal diagnosis

SNPs homozygous in both parents with two different genotypes and heterozygous in the fetal were selected for fetal fraction calculation, using the following formula (Lo et al., 2010): $f=\frac{\sum 2p}{\sum \left(p+q\right)}$ , where *p* is the count of paternal specific alleles and *q* is the count of maternal alleles in the plasma shared by both maternal and fetal genomes.

The informative SNPs in the target region were then selected to construct the paternal haplotype based on the genotypes of grandparents. As the fetus’s father and grandmother carry the pathogenic D4Z4 allele, SNPs which were heterozygous in the father, and heterozygous in one of the grandparents and homozygous in the other were selected (Table 1). The paternal haplotype transmitted from the affected grandmother was defined as the pathogenic haplotype (hap0), and the normal haplotype was defined as hap1. The fetal inheritance of paternal haplotypes was then deduced by Hidden Markov Model (HMM) (Tanaka et al., 1993; Meng et al., 2014; Saba et al., 2017).

**TABLE 1:** Paternal informative SNP selection for haplotype construction.

No.	I-1	I-2	II-2	II-3
1	0/1	0/0	0/1	0/0
2	0/1	0/0	0/1	1/1
3	0/1	1/1	0/1	0/0
4	0/1	1/1	0/1	1/1
5	0/0	0/1	0/1	0/0
6	0/0	0/1	0/1	1/1
7	1/1	0/1	0/1	0/0
8	1/1	0/1	0/1	1/1

Note: three conditions need to be met: 1. SNPs, which were heterozygous in the father; 2. SNPs, which were heterozygous in one of the grandparents; 3. homozygous in the other.

## Results

### Pedigree analysis and clinical manifestations

**FIGURE 1:**
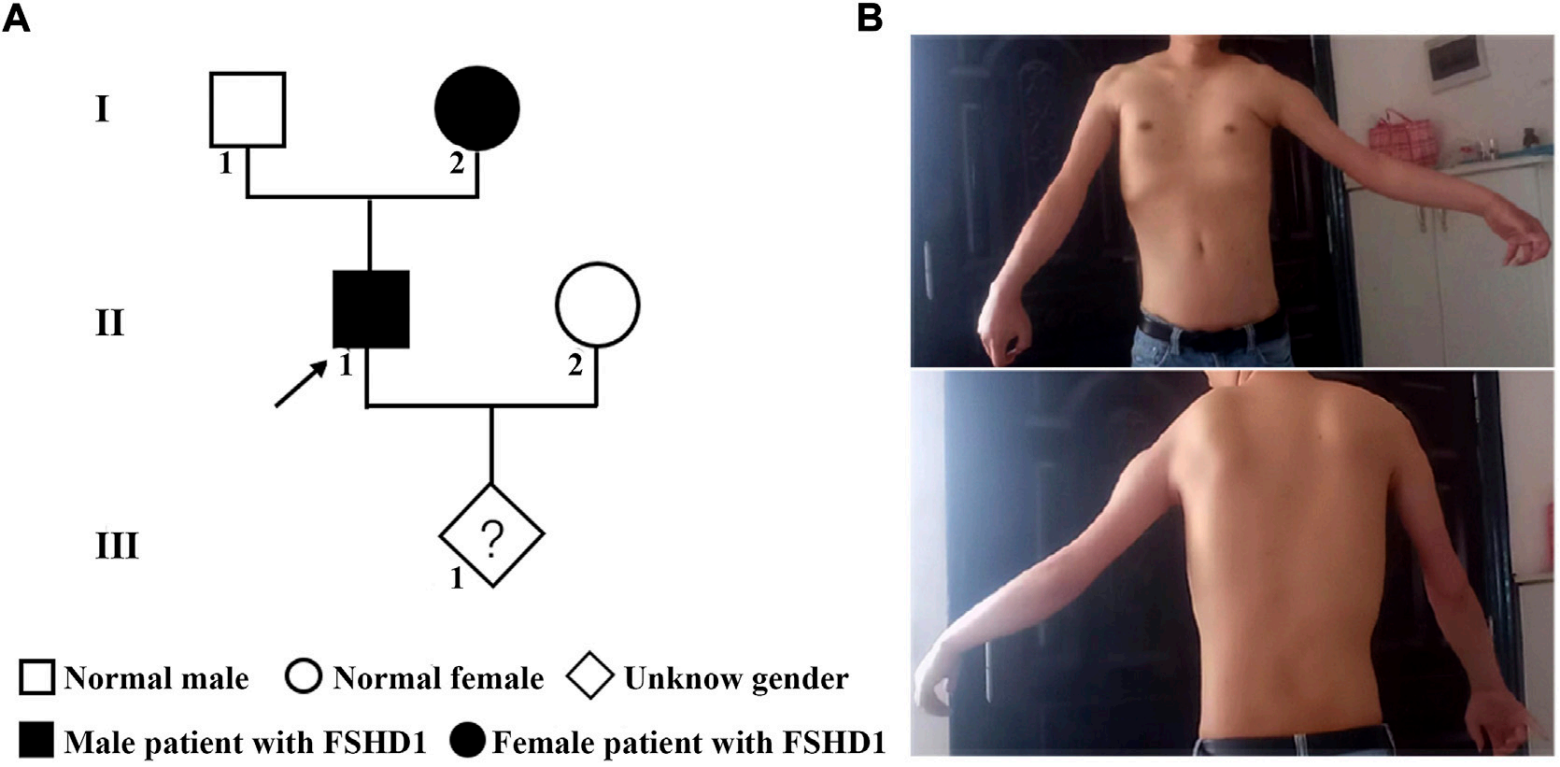
The pedigree and phenotype of the family with FSHD1. **(A)** The genetic map of the FSHD1 family. **(B)** Typical upper body profile of the patient with facioscapulohumeral muscular dystrophy. The proband showed asymmetric limitation of shoulder abduction, pectoral muscle atrophy and asymmetric scapular winging.

The family history showed a dominant inheritance pattern of disease in the family (Figure 1A). The proband (II-1), a 30 years old man, had typical upper body profiles of facioscapulohumeral muscular dystrophy, such as asymmetric limitation of shoulder abduction, pectoral muscle atrophy, and jutting upwards of the scapula on the left on attempted shoulder abduction and symmetric scapular winging (Figure 1B). The proband’s mother (I-2) also showed similar manifestations. The musculoskeletal-related tests showed a high level of serum CK-MB (26.86 ng/ml) and myogenic lesions of limbs in the proband.

### Bionano optical mapping identified the pathogenic D4Z4 allele in the proband

**FIGURE 2:**
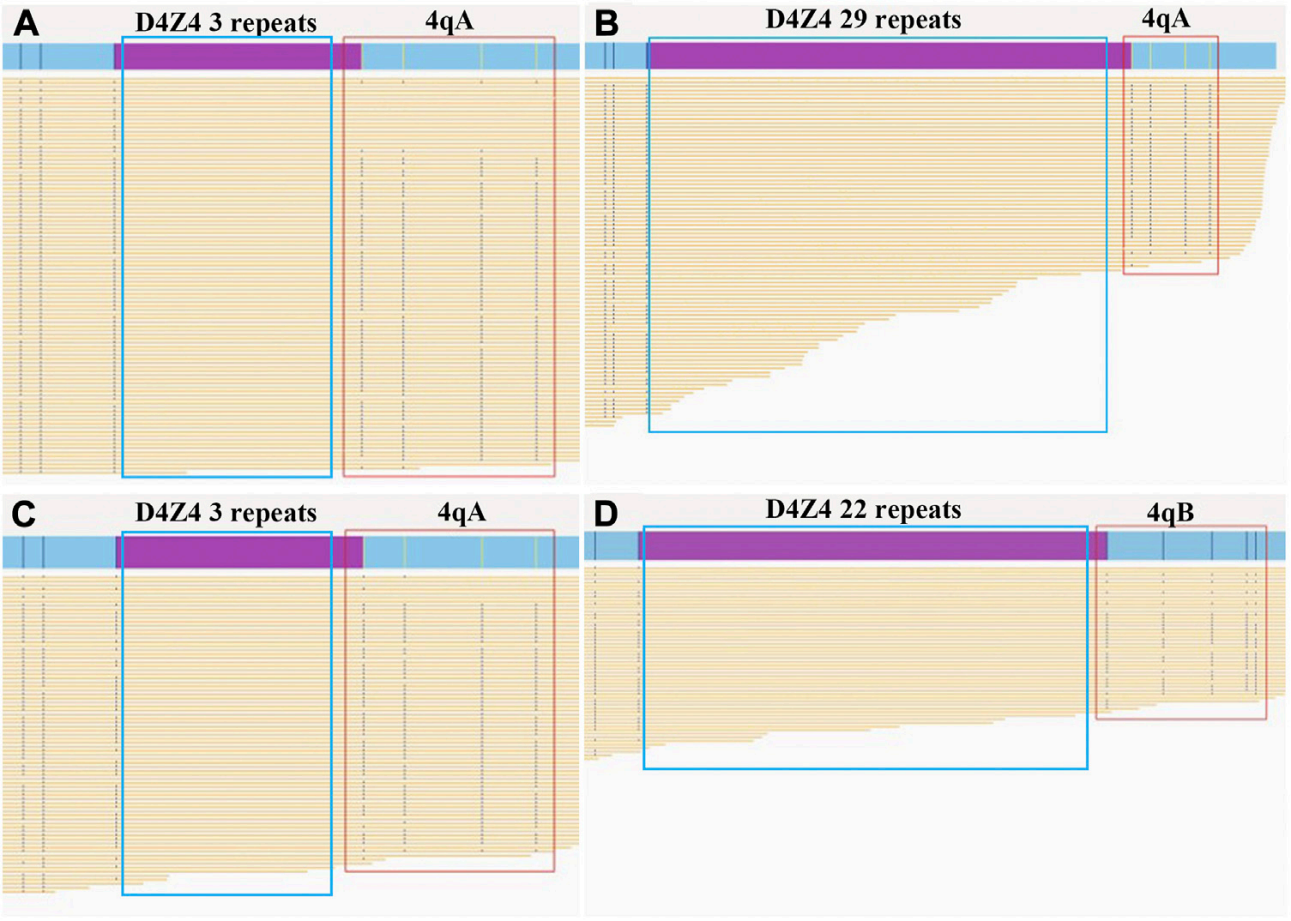
Bionano optical maps and genetic diagnosis of the father and fetus. The purple bars show the D4Z4 repeat array in the chromosomal region 4q35. The red boxes indicate the 4qA or 4qB allele. The yellow lines show the reads in the target region produced by Bionano optical mapping. **(A)** The pathogenic allele (3 D4Z4 repeats with 4qA allele type) in the affected father. **(B)** The normal allele (29 D4Z4 repeats with 4qA allele type) in the affected father. **(C)** The pathogenic allele (3 D4Z4 repeats with 4qA allele type) in the fetus. **(D)** The normal allele (22 D4Z4 repeats with 4qB allele type) in the fetus.

BOM was performed to reveal the potential genetic cause of FSHD in the proband. The data amount from gDNA of the proband was 622 Gb with a depth of 180 X. We identified three D4Z4 repeats coexisted with 4qA allele in the 4q35 region (Figures 2A,B).

### Haplotype-based NIPD of FSHD1

**FIGURE 3:**
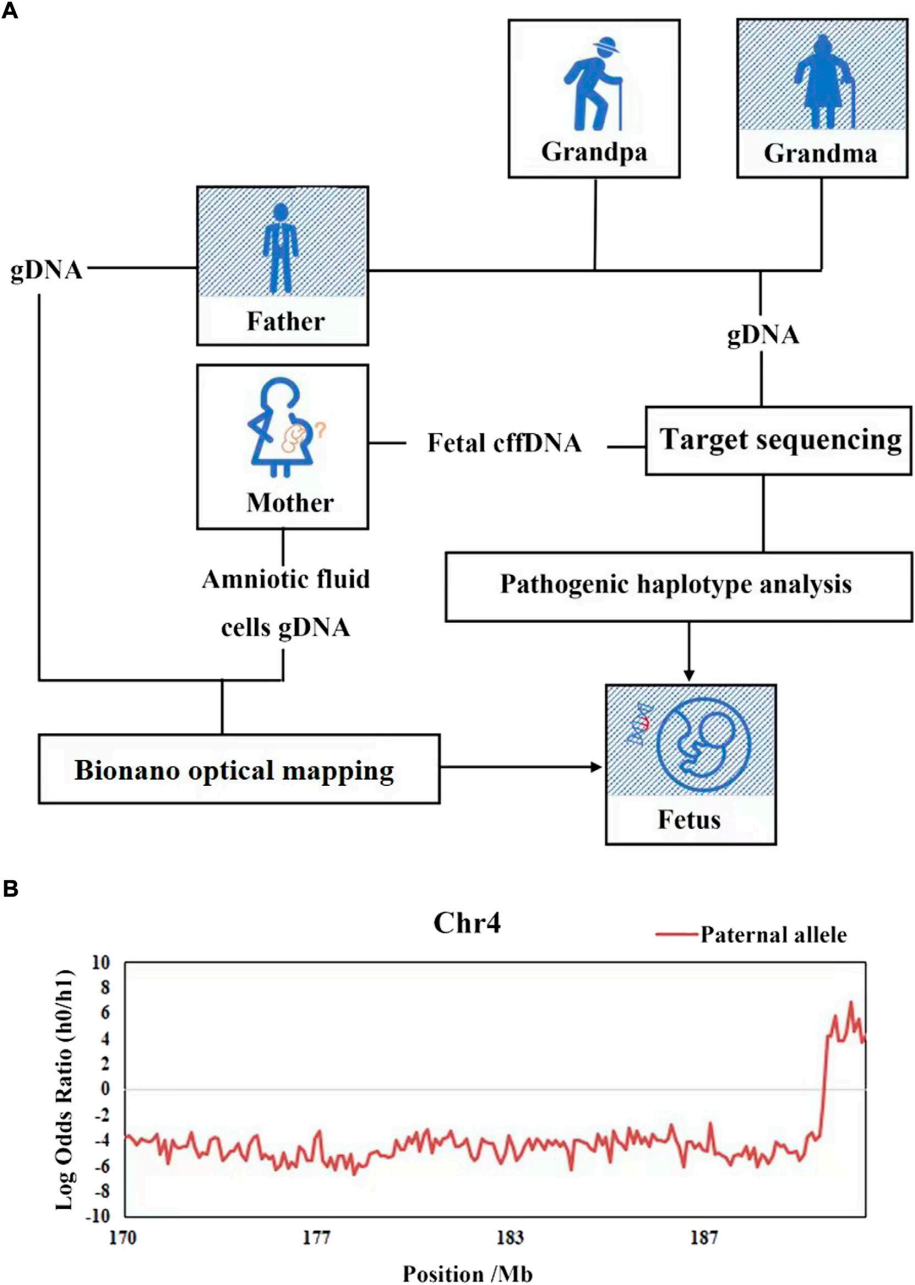
Haplotype-based NIPD of the fetus of family FSHD1. **(A)** Workflow of NIPD of FSHD1. **(B)** The HMM-based prediction of recombination and the predicted fetal haplotype. The *x*-axis represents loci on target area, the *y*-axis represents the logarithmic values of the odds ratios of fetal inheritance of the paternal haplotypes, the red lines represent the paternal haplotype of fetal inheritance. The lines above zero (gray line) indicate that the pathogenic allele was inherited (Hap0), while the lines below zero mean the normal allele is inherited (Hap1), there was a recombination event been detected at the end.

The fetal fraction of cffDNA in plasma was ranged from 5.72% to 20.78% with a median of 15.58% in this family, while the sequencing error rates were estimated to be 6e-4. According to Mendel’s law of inheritance, we deduced the paternal haplotype firstly from the fetal grandparents, and further to clear the genetic relationship of FSHD1 within this family (Figure 3A). 114 SNP loci were used to construct paternal haplotype, all of which located in the upstream of D4Z4 repeat array.

On the basis of the parental haplotype data and the maternal plasma DNA sequencing data, the fetal haplotype was inferred using HMM, the hidden states were the fetal genotypes and the observed states were mixtures of the sequencing depths of the maternal and fetal genotypes in the plasma. The number of paternal informative SNPs on target area for fetal haplotype construction was 114, the nearest one to D4Z4 repeat was located 0.08 Mb upstream. The HMM-based prediction of recombination and the predicted fetal haplotype are shown in Figure 3B. We defined haplotype 0 (Hap0) as the pathogenic haplotype and haplotype 1 (Hap1) as the normal haplotype. The fetus (III-1) was predicted to inherit Hap1 (the normal haplotype) from the father in the main part of this targeted area. However, there was a recombination event detected, which had taken place from chromosome coordinates 189,051,401, implicating that the fetal haplotype had switched to Hap0 (the pathogenic haplotype) on the downstream region. These results indicated that fetus was predicted to be a carrier of the retracted D4Z4 repeat number mutation.

### Bionano optical mapping of the fetal amniotic fluid cells

BOM of the fetal amniotic fluid cells were used to confirm the genotype of the fetus. The sequencing data amount from the fetal amniotic fluid cells was 511 Gb with a depth of 136 X. The fetus inherited the pathogenic 4q35 region with three D4Z4 shortened repeats and the 4qA allele from his father, the normal 22 D4Z4 repeats and 4qB allele from its mother (Figures 2C,D). This result was consistent with the result of cffDNA-based haplotyping.

## Discussion

FSHD1 is one of the most common muscular dystrophies characterized by progressive asymmetrical weakness of the muscles of face and shoulder girdle. The incomplete penetrance of the disease and its complicated genetic mechanisms present challenges for genetic counselling (Wohlgemuth et al., 2018). The disease is associated with a contracted D4Z4 on chromosome 4q35, which is a highly similar 3.3 kb single repeat unit whereas chromosome 10 harbors nearly identical D4Z4 repeats. The high similarity and GC content sequences makes the molecular diagnosis of FSHD1 more difficult. Direct next generation sequencing is not appropriate for detecting large repeat array because of its short-read nature. Southern blot is a traditional method to diagnose FSHD1 by measuring the length of D4Z4 array and estimating the number of repeats, although it is process-complicated, labor- and time-intensive (Deidda et al., 1996; Ehrlich et al., 2006; Vincenten et al., 2022). Third generation sequencing is qualified for molecular diagnosis of FSHD1 in advantage of its ultra-long read (Morioka et al., 2016; Mitsuhashi et al., 2017), however, it requires high molecular weight DNA. BOM is another technique for invasive prenatal diagnosis of FSHD1 gene based on high molecular weight DNA. Due to the length distribution of fetal DNA fragments is about <140bp, none of the southern blot, third generation sequencing and BOM is suitable for in the context of NIPD of FSHD1.

Prenatal diagnosis for pregnant carries gives reproductive choice and guides delivery management to ensure newborn safety (Huq and Kadir, 2011). BOM has been used for invasive prenatal diagnosis of FSHD1 gene. NIPD provides an attractive choice for pregnant carries who are reluctant to undergo invasive testing. In recent years, NIPD for dominant or recessive monogenic diseases has been reported quite many times, while it has not been reported yet to be applied in NIPD of FSHD1 in clinical practice.

In this study, BOM was first used to provide accurate molecular diagnosis for the proband with FSHD. Prenatal diagnosis was performed in the family *via* noninvasive way and also confirmed by invasive Bionano method in amniocentesis DNA test. The pregnant woman received amniocentesis at weeks of 19 for BOM analysis. On the other hand, haplotype-based NIPD was performed on the cfDNA. As expected, two methods indicated the same results that the fetus would likely be affected for FSHD1. Compared with the conventional invasive prenatal diagnosis, such as chorionic villus biopsy and amniocentesis, haplotype-based NIPD required only maternal peripheral blood, and 6 weeks earlier than chorionic villus biopsy, 10 weeks earlier than amniocentesis, it provided safer and earlier options for families with high risk of FSHD1. Furthermore, haplotype-based strategy relies on SNPs analysis, which eliminates the need for direct sequencing of low complexity regions and limitation of the short length of cfDNA. Theoretically, it is applicable for most inherited diseases which are caused by the dynamic mutations, such as Huntington’s disease (Bustamante-Aragones et al., 2012), fragile X syndrome, spinocerebellar ataxia *etc.*

Family-based haplotyping usually need a complete pedigree, several members including the first child or a grandparents carried variation at least, however, it is not always satisfied in clinical practice. In this family, in view of the fetus is the firstborn, we need to deduce the genotype through the genotyping of grandparents. Therefore, it may bring challenges to sample collection and hinder the analysis process if no family relatives or incomplete family members are presented. In addition, because of the analysis method depends a precise quantification of alleles ratio, the accuracy of NIPD would be restricted if fetal fraction is at a very low level (e.g. < 5%). Meanwhile, it is more complex to diagnose when mother carrying the pathogenic variant since doing so requires maternal alleles to be distinguished from fetal ones. The haplotype-based method should be systematically evaluated in a large population. But it could serve as an accurate choice for NIPD of FSHD or other single-gene disorders in which the culprit genes localized in the low-complexity region or have homologous or pseudogene, exerting great difficulties to be detected in cfDNA.

## Conclusion

In summary, we presented that haplotype-based NIPD combined with BOM provide a new approach for rapid and accurate prenatal diagnosis of FSHD1 families.

## Data Availability

The data presented in the study are deposited in the NCBI BioProject repository, accession number PRJNA890421.
